# Long noncoding RNAs in osteosarcoma via various signaling pathways

**DOI:** 10.1002/jcla.23317

**Published:** 2020-04-06

**Authors:** Jinming Han, Xiaohan Shen

**Affiliations:** ^1^ Ningbo NO.6 Hospital Ningbo China; ^2^ Ningbo Diagnostic Pathology Center (Shanghai Cancer Center Ningbo Pathology Center) Ningbo China; ^3^ Ningbo Medical Center Lihuili Hospital Ningbo China

**Keywords:** lncRNAs, osteosarcoma, signaling pathway

## Abstract

Osteosarcoma is one of the most commonly seen bone malignancies with high incidence rate in both children and adults. Although the regulatory network of osteosarcoma has been greatly concerned for years, the mechanisms regarding its oncogenesis and development are still not clear. Recent discoveries have revealed that long noncoding RNAs (lncRNAs) play a crucial role in the development, progression, and invasion of osteosarcoma. Deregulated expression of lncRNAs has been found to participate in the regulation of various signaling transduction pathways in osteosarcoma. This review summarized roles of lncRNAs in the pathogenesis, development, and potential therapeutic of osteosarcoma via different signaling pathways. For examples, MALAT1, CCAT2, FER1L4, LOXL1‐AS1, OIP5‐AS1, PVT1, DBH‐AS1, and AWPPH regulate PI3K/Akt signaling; AWPPH and BE503655 regulate Wnt/β‐catenin signaling; NKILA and XIST regulate NF‐κB signaling; MEG3 and SNHG12 regulate Notch signaling; FOXD2‐AS1 and LINK‐A regulate HIF‐1α signaling; GClnc1 and HOTAIR regulate P53 signaling; ZFAS1, H19, and MALAT1 regulate MAPK, Hedgehog and Rac1/JNK signaling, respectively.

## 1 INTRODUCTION

Osteosarcoma (OS) is one of the most commonly seen malignant bone tumors, which has higher incidence in both children and teenagers with lung metastasis.^1^, ^2^ Three main strategies, namely surgical resection, chemotherapy, and radiotherapy are applied for patients currently.^3^ Neoadjuvant chemotherapy combined with surgical treatment has been reported to improve the 5‐year survival rate to nearly 70% in localized OS over the past three decades, however, chemoresistance to these drugs is still the main obstacle for curing this malignancy,^1^, ^4^ thereby leading to poor prognosis, high recurrence, and metastasis. Therefore, identification of effective biomarkers for pathogenesis, prognosis, and metastasis is essential for OS patients.^5^ A variety of potential biomarkers have been identified in recent years, which could widely regulate the oncogenesis, prognosis, and metastasis of OS. Nevertheless, the detailed molecular regulatory mechanisms of OS needs further exploration.^6^, ^7^

In the last few decades, ground‐breaking progress in transcriptional regulators of mammalian genome, known as “non‐coding RNAs” (ncRNAs), has become a major concern of molecular biological research worldwide. Although initially considered to be transcriptional noise or so‐called “Junk” for lack of coding potential, numerous ncRNAs have been found to play pivotal roles in cellular processes of different diseases, including cancer.^8^, ^9^, ^10^ Regulatory ncRNAs are mainly categorized into two classes based on their length: Short ncRNAs which are less than 200 nucleotides in length represented by microRNAs (miRNAs), and long ncRNAs (LncRNAs) with the length larger than 200 nucleotides. LncRNAs comprise long intergenic ncRNAs (lincRNAs), intronic lncRNAs, antisense lncRNAs, and competing endogenous RNAs (ceRNAs) referring to multiple regulatory mechanisms of gene expression.^10^

Multiple mechanisms have involved in the lncRNAs regulatory effects of gene expression. For instance, lncRNAs may act as epigenetic regulators, signals for transcription promotion, decoys for transcription repression, or scaffolds to form ribonucleoprotein complexes by interacting with proteins. LncRNAs can modulate the stability and translation of mRNAs, act as precursors of miRNAs, as well as regulating the distribution of miRNAs et al In general, lncRNAs have been found to regulate gene expression at transcriptional and/or post‐transcriptional levels.^11^, ^12^ Accumulating evidence indicates that aberrant expression of lncRNAs may be associated with various cancers including OS, and serve as prognostic, diagnostic, and therapeutic biomarkers in OS.^13^, ^14^, ^15^, ^16^, ^17^, ^18^, ^19^, ^20^

## 2 LncRNAs IN OS VIA DIFFERENT SIGNALING PATHWAYS

Cell signaling plays a crucial role in cellular process and has a great impact on the modulation of gene expression in various cancers. Emerging evidence suggests that lncRNAs are concerned with OS through a variety of signaling pathways. For example, lncRNA HOTAIR promotes the proliferation and invasion of OS cells via the AKT/mTOR signaling pathway.^21^ The metastasis‐associated lung adenocarcinoma transcript 1 (MALAT1) acts as an oncogene for OS cells metastasis via the PI3K/AKT pathway.^22^, ^23^ It is critical to investigate the association between signaling pathways and lncRNAs in OS for developing novel strategies of early diagnosis and treatment. This review will present an overview of lncRNAs in the oncogenesis, development, and potential treatment of OS through different signaling pathways.

### 2.1 LncRNAs and PI3K/Akt signaling in OS

Numerous reports confirm the role of AKT whose activity usually involves various regulators in cancer. Among them, the most important one is PI3K, known as a positive Akt regulator. The PI3K/Akt signaling pathway plays important roles in different cellular processes, such as tumor cell growth, apoptosis, migration, and invasion.^11^, ^24^ Researchers discovered that lncRNAs may function by targeting different components of the PI3K/Akt pathway with a post‐transcriptional regulation mechanism.^11^ MALAT1 is one of the first recognized lncRNAs associated with various cancers^25^ which acts as a therapeutic target in OS.^26^ Although without translation into protein in vivo, MALAT1 can modulate the alternative splicing of pre‐mRNAs by regulating serine/arginine splicing factors. MALAT1 is also involved in the development and invasion of tumor cells. Dong et al^23^ reported that MALAT1 was overexpressed in OS tissues, and its downregulation significantly inhibited cell proliferation, migration, and invasion in vitro as well as metastasis in vivo. Besides, effects of knockdown of MALAT1 on two signaling pathways ERK/mitogen‐activated protein kinase (MAPK) and PI3K/Akt pathways were investigated and found that downregulation of MALAT1 reduced the levels of phosphorylated PI3K p85α and Akt, indicating that MALAT1 may induce proliferation and metastasis of OS cells via the PI3K/Akt pathway. Another study demonstrated that MALAT1 acts as a post‐transcriptional regulator which upregulates RET expression by binding miR‐129‐5p, thereby increasing expression of RET protein in the downstream of PI3K/Akt pathway.^22^

Colon cancer‐associated transcript 2 (CCAT2), located in the hotspot of the tumor‐related rs6983267 SNP, has been associated with increased predisposition for several cancers.^27^, ^28^, ^29^, ^30^ Overexpression of CCAT2 has been found in a wide range of cancers, in which it promotes the proliferation, invasion, migration, and survival of cancer cells. CCAT2 is considered to promote the occurrence and development of different malignancies through various pathways, such as the Wnt/TGF‐b^29^, ^31^ and GSK3b/b‐catenin signaling pathways.^32^ Liu et al^33^ revealed that CCAT2 expression is highly expressed in OS tissues, while its knockdown inhibits proliferation and mobility of OS cells in vitro. In addition, CCAT2 can inactivate the PI3K/Akt and MAPK signaling pathways, whereas a miR‐200b inhibitor partially offsets the effect of si‐CCAT2. These observations indicate that CCAT2 might play an important function in regulating OS via the PI3K/Akt and MAPK pathways. This result provides a novel diagnostic marker for OS.

The long noncoding RNA Fer‐1‐like protein 4 (FER1L4), initially identified in gastric cancer, is involved in tumorigenesis of numerous cancers such as hepatocellular carcinoma and glioma.^34^, ^35^ Recently, FER1L4 has been recognized as a prognostic biomarker in OS^36^ and the expression was found decreased in OS tissues and cell lines.^24^ Additionally, researchers revealed the effect of FER1L4 on epithelial‐to‐mesenchymal (EMT) and discovered that its knockdown promotes cell viability, inhibits apoptosis and caspase 3 activity as well as decreasing c‐caspase 3 expression and Bax/Bcl‐2 ratio. Depletion of FER1L4 results in the upregulation of key EMT markers. Furthermore, the authors demonstrated that PI3K levels and AKT phosphorylation are increased after its knockdown, suggesting that downregulation of FER1L4 inhibits cell apoptosis and facilitates EMT via the PI3K/Akt signaling pathway.^24^

**FIGURE 1:**
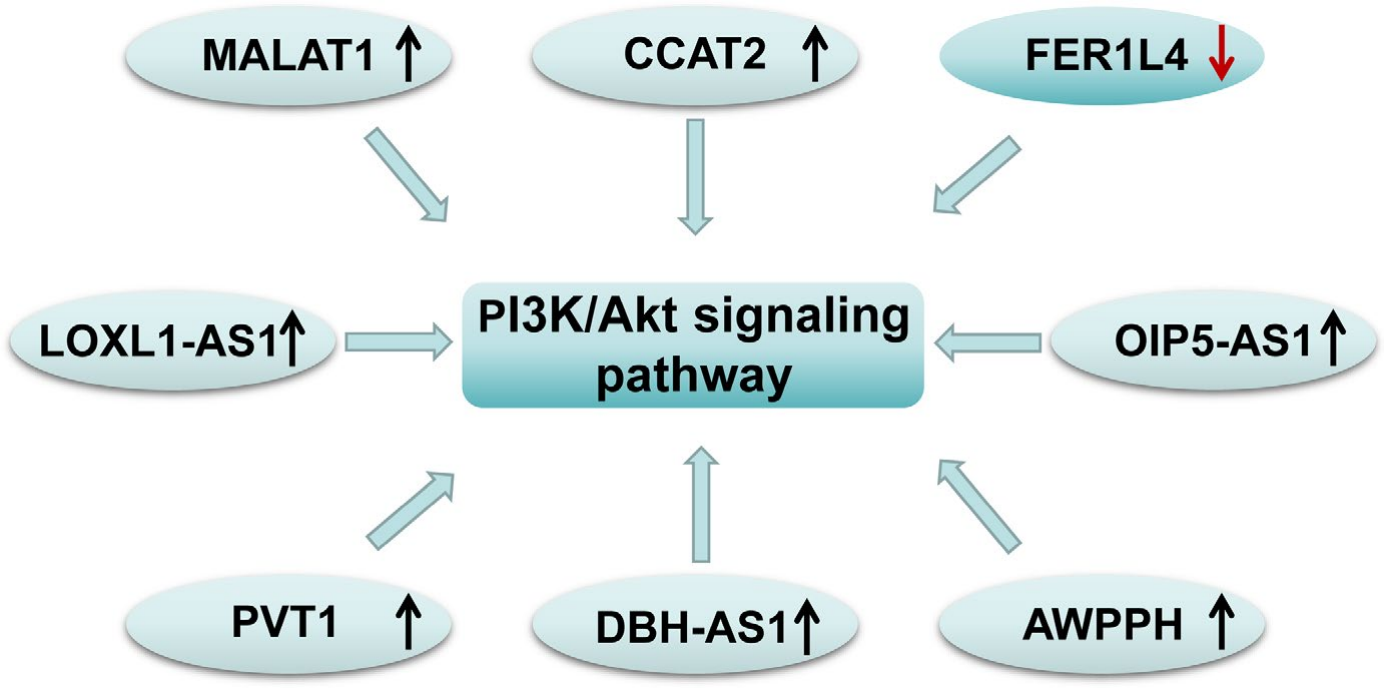
LncRNAs in osteosarcoma via PI3K/Akt signaling pathway

Other lncRNAs, such as LOXL1 antisense RNA 1 (LOXL1‐AS1), OIP5‐AS1, PVT1, DBH‐AS1, and AWPPH, have been detected in various human cancers,^37^, ^38^, ^39^, ^40^, ^41^, ^42^, ^43^, ^44^, ^45^, ^46^, ^47^, ^48^, ^49^, ^50^, ^51^, ^52^, ^53^, ^54^ which are predictive of clinical progression and poor prognosis in OS patients and function as oncogenic lncRNAs to regulate cancer progression through the PI3K‐Akt pathway.^38^, ^47^, ^51^, ^54^ LOXL1‐AS1 is encoded on the opposite strand of lysyl oxidase‐like 1(LOXL1), which was found to strongly associated with exfoliation glaucoma and exfoliation syndrome.^37^, ^38^ High expression of LOXL1‐AS1 was correlated with Enneking stage, tumor size, metastasis, histological grade, and overall survival time in OS patients. Furthermore, LOXL1‐AS1 overexpression acts as an independent poor predictor for overall survival. Knockdown of LOXL1‐AS1 dramatically inhibits OS cell proliferation, migration, and invasion through the PI3K‐Akt signaling pathway.^38^ Transcribed from the OIP5 gene on chromosome 15q15.1, OIP5‐AS1 has been found highly expressed in cisplatin resistant (CR) OS cells and its knockdown significantly reduces cisplatin resistance. Knockdown of OIP5‐AS1 suppresses the PI3K/ Akt/mTOR signaling pathway by acting as an endogenous RNA of miR‐340‐5p.^47^ PVT1 is a novel lncRNA encoded by the human PVT1 gene with oncogenic roles in numerous human cancers that has been widely studied.^47^, ^48^, ^49^, ^50^ A recent study showed that overexpressed PVT1 promotes proliferation and inhibits apoptosis in OS cells(treated with gemcitabine). Conversely, PVT1‐knockdown OS cells (treated with gemcitabine) show significantly lower survival rates as well as promoting apoptosis. Researchers also observed that PVT1 downregulates the level of miR‐152 and induces the activation of PI3K/Akt pathway. Moreover, the importance of c‐MET in the regulation of PI3K/Akt signaling by PVT1 and miR‐152 was confirmed, which indicates that PVT1 is essential in the development of chemoresistance in OS cells through the miR‐152‐dependent activation of the c‐MET/PI3K/Akt pathway.^51^ DBH‐AS1 is also a newly identified lncRNA transcribed from chromosome 9q34.^52^, ^53^ A recent study showed that OS patients with high DBH‐AS1 have remarkably shorter overall survival than patients with low DBH‐AS1. Overall, these observations indicate that DBH‐AS1 may act as a new diagnostic and prognostic biomarker for OS. Knockdown of DBH‐AS1 distinctly suppresses the proliferation of cancer cells, while its downregulation promotes apoptosis by increasing the activity of caspase 3 and caspase 9. Additionally, the study indicated that suppression of DBH‐AS1 can inhibit the activation of the PI3K/Akt pathway, which was demonstrated by examining p‐PI3K and p‐Akt expression levels.^54^ AWPPH is a novel lncRNA that has oncogenic functions in various cancers.^55^, ^56^ One study showed that AWPPH depletion suppresses OS cell migration and invasion, and AWPPH knockdown was demonstrated to induce cell apoptosis by regulating protein expressions of Bcl‐2, Bax, cleaved‐caspase‐3 as well as cleaved‐caspase‐9. Furthermore, downregulation of AWPPH decreases the expression levels of p‐PI3K and p‐Akt significantly, suggesting that AWPPH may play an oncogenic role through the PI3K/Akt pathway.^57^ (Figure 1).

### 2.2 LncRNAs and Wnt/β‐catenin signaling in OS

Wnt pathway mutations have been frequently observed in cancer. Aberrant activation of Wnt/β‐catenin signaling is closely related to various cancer types as it controls a number of oncogenes, which leads to cell proliferation and tumorigenesis.^11^ Recently, lncRNAs have been found to regulate gene expressions in OS cells through different pathways due to its complex molecular mechanisms. AWPPH is such an example. In another study, researchers confirmed that AWPPH is positively related with tumor size or metastasis in OS.^58^ Furthermore, they confirmed that AWPPH promotes OS development through inhibiting miR‐93‐3p, a tumor suppressor in other cancers such as clear cell renal cell carcinoma and breast cancer.^59^, ^60^ In addition, the study showed that miR‐93‐3p targets FZD7, and miR‐93‐3p inhibition by AWPPH promotes the expression of FZD7, which leads to tumorigenesis and acts as a crucial regulator in the Wnt signaling,^61^ thereby indicating that AWPPH activates Wnt/β‐catenin signaling pathway via the miR‐93‐3p/FZD7 axis in OS development.^58^

Numerous dysregulated lncRNAs have been identified by RNA‐seq, such as lncRNA BE503655, whose function and regulatory mechanism were studied.^62^ LncRNA BE503655 was found to be highly expressed in both OS tissues and cell lines. LncRNA BE503655 downregulation inhibits OS cell proliferation and invasion. Besides, there is a positive correlation between its expression and Enneking stage, distant metastasis as well as histological grade. Moreover, researchers performed QT‐PCR to screen the involvement of different pathways, including Wnt/β‐catenin, mTOR, and JNK signaling, and found that β‐catenin is overexpressed in OS tissues and positively related to lncRNA BE503655 expression. Upon lncRNA BE503655 knockdown in OS cells, β‐catenin mRNA level and protein expression are significantly decreased. Furthermore, some markers in the Wnt/β‐catenin pathway are also downregulated, suggesting that lncRNA BE503655 exerts its activity by regulating Wnt/β‐catenin pathways in OS.

### 2.3 LncRNAs and NF‐κB signaling in OS

Nuclear factor‐κB (NF‐κB), a widely expressed transcription factor, is one of the most significant molecules connecting inflammation with cancer. The activation of NF‐κB pathway is regulated by multiple mechanisms and plays a key role in cancer progression including cell growth, invasion, and metastasis.^63^ LncRNAs such as NKILA have been found in NF‐κB pathway to act as a scaffold for recruiting a variety of proteins in breast cancer metastasis.^11^ It also can inhibit NF‐κB activation induced by TGF‐β and act as a target for preventing breast cancer metastasis by inhibiting EMT.^64^ In hepatocellular cancer(HCC), NKILA could enhance the anticancer effect of safrole by regulating the NF‐κB signaling pathway.^65^ In a study concerning OS, researchers learned that NKILA expression in OS tissues is strikingly reduced, with its level lower in stage III tumor tissues than that in stage I‐II. Meanwhile, high expression of NKILA considerably reduces the proliferation, invasion, and migration of OS cells. NKILA expression was found to be activated by the NF‐κB pathway, which could directly block the phosphorylation of IκB by interacting with the NF‐κB/IκB complex, thereby inhibiting IKK phosphorylation and NF‐κB activation. In this study, they also found that knockdown of NKILA strikingly reduces the expressions of E‐cadherin, which mediates the loss of cell adhesion, a prerequisite for the infiltration and metastasis of tumor cells. Additionally, an NF‐κB inhibitor could reverse the effect of knockdown of NKILA on cell migration and proliferation.^66^

The lncRNA X‐inactive specific transcript (XIST) has been discovered to promote cancer development and was related to poor prognosis.^67^, ^68^, ^69^ Nevertheless, Zhang et al^70^ revealed that in OS XIST functions as a tumor suppressor. Gao et al^71^ demonstrated that targeting XIST can activate NF‐κB, p65 nuclear translocation as well as the PUMA signaling pathway. Targeting PUMA promotes cell viability and suppresses cell apoptosis in XIST shRNA transfected OS cells, demonstrating that targeting XIST suppresses cell proliferation and tumorigenesis by activating the NF‐kB and NF‐kB‐dependent PUMA signaling pathway. These findings indicated that XIST may act as a therapeutic marker for OS treatment.

### 2.4 LncRNAs and Notch signaling in OS

As a highly conserved cell signaling pathway, the Notch signaling is also essential to multiple biological processes and participates to the development of various cancers.^11^, ^72^, ^73^ Interestingly, several lncRNAs have been shown to be regulated by Notch. One of them is MEG3, a well‐studied lncRNA encoded by the MEG3 transcript of the DLK1/MEG3 locus on human chromosome.^74^ Evidence has shown that as a tumor suppressor gene, MEG3 is downregulated in a variety of malignancies and is associated with poor prognosis.^72^, ^75^, ^76^ MEG3 has a lower expression in OS tissues compared with adjacent non‐tumor ones which is also found expressed at low levels in OS cell lines. In addition, overexpression of MEG3 significantly represses the growth and migration ability of OS cells, confirming the tumor suppressive role of MEG3 in OS progression.^77^ Meanwhile, Notch signaling has been revealed closely associated with cancer cellular EMT and metastasis.^73^, ^78^, ^79^ Previous studies have proved that MEG3 is related to Notch and TGF‐β pathways.^80^ To further explore the activity of MEG3 on OS cell behavior, the Notch and TGF‐β signaling pathways were analyzed, and researchers found that MEG3 overexpression inhibited the expression of Notch1, Hes1, TGF‐β, and N‐cadherin and enhanced E‐cadherin expression.^81^

Small nucleolar RNA host gene 12 (SNHG12), a newly discovered lncRNA, has recently been found upregulated in several types of tumors including OS.^82^, ^83^, ^84^, ^85^ Zhou et al^86^ discovered that overexpression of SNHG12 was positively related to tumor grade, Enneking stage, tumor size, and metastasis as well as poor overall survival. Meanwhile, invasion and migration of OS cells were inhibited by si‐SNHG12. These findings indicate that SNHG12 may function as a prognostic factor for OS pathogenesis and metastasis. Recently, the Notch pathway has been demonstrated closely associated with OS progression and inactivation of Notch signaling may overcome drug resistance.^87^, ^88^ One study revealed that SNHG12 promoted oncogenesis and metastasis of OS by sponging miR‐195‐5p and subsequently activating the Notch2(an important receptor)‐Notch signaling pathway.^86^, ^89^

### 2.5 LncRNAs and HIF‐1α signaling in OS

As the most significant modulator in the response to hypoxia, HIF‐1α has been confirmed to regulate hypoxic gene expression via signaling transduction networks. Numerous studies have revealed the existence of lncRNAs in hypoxic tumor regions. One of them is FOXD2‐AS1, a novel lncRNA which may function as a miRNA sponge to modulate tumorigenesis. The aberrant expression of FOXD2‐AS1 has been found to correlate with HCC by silencing CDKN1B (p27) via recruiting EZH2, an important element of PRC2 (polycomb repressive complex 2 to its promoter region.^90^ Ren et al^91^ discovered that FOXD2‐AS1 expression was upregulated in OS with a positive relation to poor prognosis, indicating the oncogenic and prognostic role in OS. HIF‐1α has been learned significantly upregulated in OS and takes part in hypoxia‐induced cell proliferation, invasion, and EMT. Researchers found that HIF‐1α could bind with the promoter region of FOXD2‐AS, thereby increasing both mRNA and protein levels. Besides, FOXD2‐AS1 could inhibit p21 protein expression by recruiting EZH2 to the p21 promoter region for silencing its transcription.^91^, ^92^, ^93^, ^94^

LincRNA for kinase activation (LINK‐A) is an oncogene in breast cancer, in which it activates HIF1α to promote triple‐negative breast cancer progression.^95^ Researchers found that plasma circulating LINK‐A is high expressed in metastatic OS patients compared to non‐metastatic OS ones, suggesting that LINK‐A might be associated with the OS metastasis specifically. Besides, LINK‐A overexpression can inhibit the expression level of HIF1α, which in turn, however, has no effect on LINK‐A. Moreover, HIF inhibitor LW6 could reduce LINK‐A‐overexpressed effects on migration and invasion in cell lines. The results estimated that LINK‐A might act as an upstream activator of HIF1α and participate in the metastasis of OS by upregulating the HIF1α pathway.^96^

### 2.6 LncRNAs and P53 signaling in OS

As a tumor suppressor, P53 has been discovered mutated in more than 50% of malignancies. p53 regulation mainly refers to translation and posttranslational modifications as well as protein stability. Increasing evidence revealed that lncRNAs are involved in the p53 pathway with different mechanisms of action.^97^ Some may act as direct transcriptional targets of p53 or play a role in DNA repair.^11^ Found in gastric cancer as an oncogene,^98^ LncRNA GClnc1 is also highly expressed in OS samples which predicts poor prognosis. Moreover, GClnc1 could promote OS cells growth both in vitro and in vivo by blocking p53 to bind to the promoter region of p21 and BAX, thereby suppressing p21 and BAX expression. Therefore, GClnc1 may be a potential target in the p53 pathway and play an oncogenic role in OS.^99^

HOTAIR was the first tumor‐related lncRNA discovered to be associated with cancer metastasis and poor prognosis. In a recent study concerning OS, HOTAIR was also detected to be upregulated as an oncogene which promotes proliferation and inhibits apoptosis in OS cells. Furthermore, the expression level of p53 after silencing HOTAIR is significantly upregulated, while bcl‐2 expression is downregulated, indicating that HOTAIR may take part in the p53‐mediated apoptosis pathway in OS cells.^10^

### 2.7 Other signaling pathways

Some signaling pathways are also correlated with lncRNAs in OS but are relatively rarely found. For instance, Zinc finger antisense 1 (ZFAS1), a newly identified lncRNA, was reported to be upregulated in OS cells and promote colony formation, migration, and invasion of OS cells via activating the MAPK signaling pathway.^11^ As an imprinted gene, long noncoding RNA H19 has been found abnormally expressed and modulated by Hedgehog signaling as well as yes‐associated protein 1 (Yap1) overexpression, demonstrating that aberrant Hedgehog signaling pathway is closely associated with the pathogenesis of osteoblastic OS through Yap1 and H19 overexpression.^102^, ^103^ In addition, another study concerning MALAT1 in OS indicated that MALAT1 could induce growth of OS cell through miR‐509 (an OS growth suppressor) inhibition, thereby leading to the Rac1/JNK pathway activation of. This finding suggests the presence of a MALAT1/miR‐509/Rac1 axis which regulates OS cell proliferation and cancer development.^14^

## 3 CONCLUSIONS AND FUTURE DIRECTIONS

Most lncRNAs do not seem to be as significant as molecules of signaling transduction on their own. However, they are crucial in regulating signaling pathways which tend to be more adaptable to tumor microenvironment. One of the most significant roles of lncRNA regarding cell signaling regulation in OS is that lncRNAs might act as a scaffold through which lncRNAs can directly interact with various signaling molecules.^11^ Numerous lncRNAs have been found involved in the tumorigenesis, progression, and invasion of OS (Table 1). Some may be downregulated compared with normal tissues or cell lines, such as MEG3, NKILA, and XIST, or be upregulated, such as MALAT1. They may serve as prognostic (eg, FER1L4, LOXL1‐AS1, and SNHG12), therapeutic (eg, MALAT‐1, AS1, and PVT1), and diagnostic (eg, CCAT2 and DBH‐AS1) markers. The last group can be widely used in liquid biopsies for OS diagnosis, which allows patients to avoid invasive diagnostic methods such as tissue biopsy. As prognostic markers, lncRNAs can help prognosis prediction and guide therapy decision‐making. Considering that the therapy of OS patients has not efficiently improved in recent years, lncRNAs with therapeutic potentials may turn into promising targets in the treatment for OS patients.^13^ Although lncRNA research is still relatively limited so far, we expect that more lncRNAs involved in different signaling pathways in OS will be discovered and illuminated in the future. Further studies of these lncRNAs will focus on the interaction mechanisms of lncRNAs and signaling molecules, as well as how they impact OS tumorigenesis and progression.

**TABLE 1:** LncRNAs involved in the pathogenesis and progression of osteosarcoma

Signaling pathways	LncRNAs	Expression level	Role	Clinical value	Related references
PI3K/Akt	MALAT1	High	Oncogene	Therapeutic	25‐27
	CCAT2	High	Oncogene	Diagnostic	34
	FER1L4	Low	Tumor suppressor gene	Prognostic	24, 37
	LOXL1‐AS1	High	Oncogene	Prognostic	39
	OIP5‐AS1	High	Oncogene	Therapeutic	48
	PVT1	High	Oncogene	Diagnostic	52
	DBH‐AS1	High	Oncogene	Diagnostic/prognostic	55
	AWPPH	High	Oncogene	Prognostic	58
Wnt/β‐catenin	AWPPH	High	Oncogene	Prognostic	59
	BE503655	High	Oncogene	Unknown	63
NF‐κB	NKILA	Low	Tumor suppressor gene	Unknown	66
	XIST	Low	Tumor suppressor gene	Therapeutic	71, 72
Notch	MEG3	Low	Tumor suppressor gene	Therapeutic	81, 82
	SNHG12	High	Oncogene	Prognostic	87
HIF‐1α	FOXD2‐AS1	High	Oncogene	Prognostic	92
	LINK‐A	High	Oncogene	Diagnostic	97
P53	GClnc1	High	Oncogene	Prognostic	100
	HOTAIR	High	Oncogene	Therapeutic	101
MAPK	ZFAS1	High	Oncogene	Diagnostic/therapeutic	102
Hedgehog	H19	High	Oncogene	Unknown	103, 104
Rac1/JNK	MALAT1	High	Oncogene	Therapeutic	105

## ETHICAL APPROVAL

This assay was reviewed and approved by the Ethics Committee of Ningbo NO.6 Hospital. All participants signed written informed consent documents.
